# Comprehensive transcriptomic analysis of Tibetan Schizothoracinae fish *Gymnocypris przewalskii* reveals how it adapts to a high altitude aquatic life

**DOI:** 10.1186/s12862-017-0925-z

**Published:** 2017-03-09

**Authors:** Chao Tong, Tian Fei, Cunfang Zhang, Kai Zhao

**Affiliations:** 1 Key Laboratory of Adaptation and Evolution of Plateau Biota, Northwest Institute of Plateau Biology, Chinese Academy of Sciences, Xining, 810001 China; 2 Laboratory of Plateau Fish Evolutionary and Functional Genomics, Northwest Institute of Plateau Biology, Chinese Academy of Sciences, Xining, 810001 China; 3 Qinghai Key Laboratory of Animal Ecological Genomics, Northwest Institute of Plateau Biology, Chinese Academy of Sciences, Xining, 810001 China; 4 University of Chinese Academy of Sciences, Beijing, 100049 China

**Keywords:** Adaptation, Comparative genomics, Tibetan Schizothoracinae fish

## Abstract

**Background:**

Understanding the genetic basis of adaptation to high altitude life is of paramount importance for preserving and managing genetic diversity in highland animals. This objective has been addressed mainly in terrestrial fauna but rarely in aquatic animals. Tibetan Schizothoracinae fish is the ideal model system in evolutionary biology, carrying key insights into evolutionary genetics of speciation and adaptation at high altitude. *Gymnocypris przewalskii* is the newly formed Schizothoracinae fish species in the Tibetan Plateau, inhabits chronic cold, extreme saline and alkaline aquatic environment in Lake Qinghai, thus evolving the unique genomic signatures to adapt extremely severe environments.

**Results:**

To characterize its genomic features, we assembled *de novo* transcriptome of *G. przewalskii* from Lake Qinghai. Intriguingly, by comparative genomic analyses of *G. przewalskii* and 8 other fish species, we identified potential expansions in gene families related to energy metabolism, transport and developmental functions, possibly underlying the adaptation to these environmental stresses. Through comprehensive molecular evolution analyses, we found that sets of genes controlling mitochondrion, ion homoeostasis, acid-base balance and innate immunity show significant signals of positive selection. Compared to previous studies on highland fishes, we failed to identify any positively selected genes related to hypoxia response.

**Conclusions:**

Our findings provide comprehensive insights into the genetic basis of teleost fish that underlie their adaptation to extreme high altitude aquatic life on the Tibetan Plateau.

**Electronic supplementary material:**

The online version of this article (doi:10.1186/s12862-017-0925-z) contains supplementary material, which is available to authorized users.

## Background

It is an interest for both evolutionary biologists and ecologists to understand how wildlife adapts to environment at high attitude [1, 2]. With the average elevation approximately 4,000 m above sea level (a.s.l.) [3, 4], the Tibetan Plateau (TP), imposes extremely inhospitable environmental challenges to all the native creatures [2, 5]. Many native Tibetan organisms have developed unique morphological, physiological and genetic features to tolerate harsh living conditions [6]. Recent studies employing genome-wide approaches mainly focused on the hypoxia and metabolic adaptation of Tibetan terrestrial animals, including yak [2], Tibetan antelope [4], ground tit [3], Tibetan Mastiff [7], Tibetan dog [8], and Tibetan Chickens [9]. Nevertheless, little is known about the adaptive mechanisms of Tibetan aquatic animals to water environment. Schizothoracinae fishes, the predominant fish fauna in the TP, evolved specific genetic and phenotypic characteristics to adapt the extreme aquatic environments, such as chronic cold, high UV and PH value. Understanding of the genetic foundation of Schizothoracinae fishes will shed novel lights on the highland adaptation of Tibetan wildlife.

**Fig. 1:**
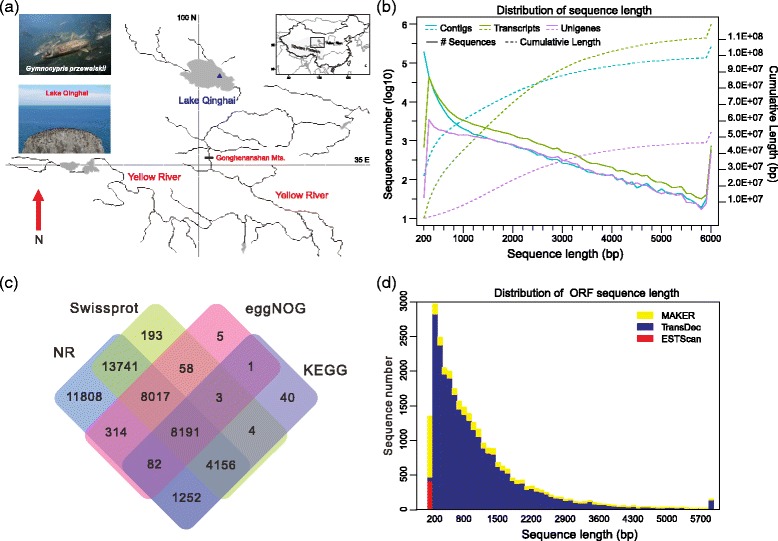
The sampling site and *G. przewalskii* transcriptome annotation. **a** The sampling map was created using the ArcGIS v10.1 (ESRI, CA, USA) and Adobe Illustrator CS5 (Adobe Systems Inc., San Francisco, CA). The blue triangle represented the sample site. Photos of *G. przewalskii* and Lake Qinghai are taken by Dr. Chao Tong. **b** Sequence number distribution and cumulative length of contigs, transcripts and unigenes. **c** Venn diagram showed shared and distinct genes under the annotations of NR, Swiss-Prot, eggNOG and KEGG databases. **d** Sequence number distribution of unigene ORF annotated by MAKER, TransDec and ESTScan

Tibetan naked carp (*Gymnocypris przewalskii*, family Cyprinidae) is one of the best characterized Schizothoracinae fish species in the TP, and it serves as an ideal model in evolutionary biology [10–14]. Using *G. przewalskii* as a research model, key genes in speciation and adaptation were identified [10–12, 15]. Unlike other broadly distributed Schizothoracinae fish species, *G. przewalskii* inhabits saline and alkaline lake (Lake Qinghai), but also survives in freshwater of connective rivers during the spawning migration (Fig. 1a). As the largest salt lake in China, Lake Qinghai is highly saline (up to 13‰) and alkaline (up to pH 9.4) water environment, a typical salt lake with unusually high sodium, potassium and magnesium concentration [16, 17]. Lake Qinghai used to be freshwater and connected to the Yellow River, while during the late well-known geological events “Gonghe Movement” (15 mya), Lake Qinghai was separated with the upper reaches of the Yellow River [17, 18]. During the early to late Holocene, *G. przewalskii* has gradually evolved from the freshwater fish to tolerate high salinity and alkalinity [17, 19]. In addition to high salinity and alkalinity, *G. przewalskii* survives in low temperature and hypoxic environment in Lake Qinghai [20–22]. Because of the unique evolutionary history in Lake Qinghai at high altitude, *G. przewalskii* provides an exceptional model to investigate the genetic mechanisms underlying adaptation to extreme aquatic environments in the TP.

Recent progresses in sequencing technologies and bioinformatics offer a great opportunity to study the transcriptomes of non-model organisms without reference genomes [23, 24]. Comparative transcriptomic analysis approaches have considerable impact on evolutionary biology and facilitate investigation of the genetic basis of evolution and adaptation. An additional advantage of transcriptomic study is its successful application in polyploidy organisms to obtain massive protein-coding genes and molecular markers [25, 26]. *G. przewalskii* is recorded as a tetraploid without a reference genome [18], therefore, we applied the comparative transcriptomic analysis to understand the genetic forces of its adaptation to the aquatic environment in Lake Qinghai. In current study, we present the first reference transcriptome of *G. przewalskii*, and characterized its genetic features relative to other available fish genomes. We also conducted multiple evolutionary analyses to uncover the potential genetic mechanisms of highland adaptation in fishes.

## Methods

### Sample collection and ethics statement

All animal experiments were approved by the Animal Care and Use Committees of the Northwest Institute of Plateau Biology, Chinese Academy of Sciences. Eight adult Tibetan naked carp individuals (four males and four females) were collected from Lake Qinghai (37°03′N, 100°26′E, Fig. 1a) using gill nets. All individuals were classified based on the gender and dissected after anesthesia with MS-222 (Solarbio, Beijing, China). Tissues from gill, kidney, brain, heart and liver from each individual were collected and immediately stored in liquid nitrogen at -80 °C.

### RNA extraction

**Fig. 2:**
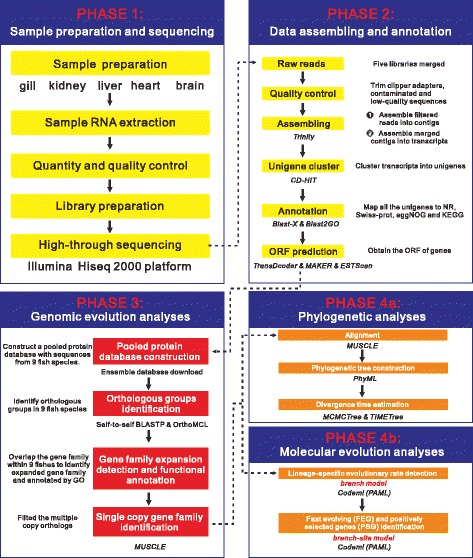
The flowchart represents four main phases in comparative transcriptome analyses process: (1) Sample preparation and sequencing; (2) Data assembling and annotation; (3) Genomic evolution analyses; (4) Phylogenetic and molecular evolution analyses

Total RNA was isolated from each eight individuals using TRIzol reagent (Invitrogen, Carlsbad, CA) according to the manufacturer′s protocol. The quantity and quality of total RNA were measured using an Agilent 2100 bioanalyzer (Agilent Technologies, Palo Alto, CA) and gel electrophoresis. Equal amount of RNA from eight individual of same tissue was pooled to construct transcriptome library independently (five libraries), and was sequenced with an Illumina HiSeq™ 2000 platform (Fig. 2).

### Reference transcriptome assembly

The raw reads from five libraries were preprocessed to remove clipped adapter sequences, contaminated sequences, low quality reads (Q < 20). All clean reads were assembled using *Trinity* software [27] with default parameters. Contigs from each assembly libraries were performed with CD-HIT [28]. Contigs from the five libraries were merged and assembled into transcripts, and non-redundant unique transcripts as long as possible were clustered into unigenes (note: unique gene), with a minimum overlap length of 200 bp (Fig. 2). The clustered unigene sequences were first aligned to four public databases with a Blast-X search (E-value cutoff of 1 × 10^−10^), including NCBI non-redundant (NR), Swiss-Prot, eggNOG and Kyoto Encyclopedia of Genes and Genomes (KEGG). Gene ontology (GO) terms were obtained from NR hits using Blast2GO software [29] with default parameters. Next, two programs *TransDecoder* (http://transdecoder.github.io/) and MAKER [30] were applied to obtain the Open reading frames (ORFs) of genes (Fig. 2). The remaining unigenes which can not be aligned to any protein database were scanned using ESTScan software [31], producing predicted coding region and direction. Finally, after removing the CDSs which length was shorter than 150 bp, all eligible CDSs unigenes were translated into amino acid (aa) sequences with standard codon table.

### Orthologs identification, sequence alignment and expanded gene family annotation

Translated Tibetan naked carp (*G. przewalskii*) amino acid sequences were pooled into a protein database with sequences (length > 50 aa) from another eight fish species genome datasets (Fig. 2): zebrafish (*Danio rerio*), cod (*Gadus morhua*), cave fish (*Astyanax mexicanus*), Fugu (*Takifugu rubripes)*, Nile tilapia (*Oreochromis niloticus*), medaka (*Oryzias latipes*), spotted gar (*Lepisosteus oculatus*) from Ensembl database (release 78) and common carp (*Cyprinus carpio*) from online database (http://carpbase.org/). Next, self-to-self BLASTP was conducted for all amino acid sequences with a E-value cutoff of 1e^−5^, and hits with identity < 30% and coverage < 30% were removed. Orthologous groups were constructed from the BLASTP results with OrthoMCL v2.0.9 [32] with default settings. All the identified orthologous groups were respectively calculated, mapped and illustrated by venn diagram. Expansion of gene families was analyzed and processed using CAFE 3.1 [33]. Finally, gene ontology (GO) functional enrichment analyses for the expanded gene family were carried out by Blast2GO software [29].

### Phylogenetic tree reconstruction

After trimming multicopy genes, single copy gene families were retrieved from OrthoMCL database as described above and then used for further phylogenetic analysis (Fig. 2). Gene families containing any sequences shorter than 200 aa were removed, and amino acid sequences in each family were aligned by MUSCLE (v3.8.31) program [34] with default parameters, and corresponding CDS alignments were back-translated from corresponding amino acid sequence alignments. Next, the families were further filtered if the CDS alignment contained any taxon in which more than 50% data was missing. The remaining CDS alignments of each family were separated into 3 sets corresponding to each of three codon positions. The four super matrices (all codon positions and each codon position) were then separately assembled into supergenes using a custom Perl script. The jModeltest program [35] was used to select the best fitting substitution model according to the Akaike information criterion based on the supergenes concatenated 4D-sites. The GTR + gamma + I model was found to be the best fitting, and PhyML3.1 [36] was employed to build the maximum likelihood (ML) tree with 1,000 nonparametric bootstrap replicates (Fig. 2).

### Divergence time estimation

We generated two datasets from CDS alignments to estimate divergence time of each species. One dataset contained the first two partitions (note: partition means the codon position), including first and second codon positions of the sequences. The other dataset contained all three partitions corresponding to all three codon positions in the sequences. Divergence times were estimated under a relaxed clock model using MCMCTree program in PAML4.7 [37], with “Independent rates model (clock = 2)” and “JC69 model” selected for our calculations. MCMC process preforms 4,000,000 iteration after a burn-in of 2,000,000. Other parameters were the default settings of MCMCTree. We ran this program twice for each dataset to confirm that the results were consistent between runs. The following constraints were used for time calibrations from TIMETree [38], a public knowledge-base of divergence times among organisms, demonstrating the high reliability of this molecular clock dating strategy (Fig. 2).

//Zebrafish – Medaka, stickleback, Takifugu, Tetraodon (min 149.85 Mya; max 165.2 Mya)

//Medaka – stickleback, Takifugu, Tetraodon (min 96.9 Mya; max 150.9 Mya)

//Zebrafish, Medaka, stickleback, Takifugu, Tetraodon – toad, bird, mammal (min 416 Mya; max 421.75 Mya)

### Molecular evolution analyses

The lineage-specific evolutionary rates for each branch of nine fish species were estimated using the *codeml* program in PAML 4.7a [37] with free-ratio model (branch model). One thousand concatenated alignments constructed from 150 randomly chosen orthologs were used to estimate lineage-specific mean values of dN and dS and the dN/dS ratio (ω value) (Fig. 2).

We used branch model in *codeml* program to identify fast evolving genes (FEGs) with null model assuming that all branches have been evolving at same rate and alternative model allowing foreground branch to evolve under a different rate (Fig. 2). The likelihood ratio test (LRT) with df = 1 was used to discriminate between alternative model for each orthologs in the gene set. Multiple testing was corrected by applying false discovery rate (FDR) method implemented in R software (https://www.r-project.org/). We considered the genes as evolving with a significantly faster rate in foreground branch if FDR-adjusted *P* value < 0.05 and a higher ω values in foreground branch than background branches.

We used *codeml* program with a branch-site model [39] to identify positively selected genes (PSGs) in the Tibetan naked carp lineages, with other lineages being specified as foreground branch (Fig. 2). A LRT was constructed to compare a model that allows sites to be under positive selection (ω > 1) on the foreground branch with the null model in which sites may evolve neutrally (ω = 1) and under purifying selection (ω < 1) with a posterior probability in excess of 0.95 based on the Bayes empirical Bayes (BEB) results [40]. Finally, *P* value was calculated based on rigorous Chi-square statistic adjusted by FDR method and genes with adjusted *P* value < 0.05 were treated as candidates under positive selection.

Gene ontology (GO) functional enrichment analyses for both FEGs and positively selected genes (PSGs) were carried out by Blast2GO software [29].

### Sequence availability

Illumina sequenced read data were deposited in NCBI Sequence Read Archive as follow: experiment (SRX2347530), and runs for each tissue including gill (SRR1542352), kidney (SRR1542353), brain (SRR5019657), heart (SRR5019658) and liver (SRR5019659).

## Results

### Sequence analysis and assembly

445,582,631 raw 101-bp paired-end reads were generated by RNA-seq from five cDNA libraries, with an average of 89 million reads per library (Additional file 1: Table S1). After removing adapters and low-quality read, totally 404,479,795 clean reads were obtained from each organ’s datasets. After assembly, 30,672 unigenes were finally yielded, ranged from 201 to 24,383 bp in length, with an N50 of 3,076 bp and an average length of 1,988 bp (Additional file 1: Table S1). The length distribution of all contigs, transcripts and unigenes is shown in Fig. 1b.

### Functional annotation

To comprehensively annotate the data, all unigenes were aligned to several public databases. A total of 28,519 (89.11%) sequences were yielded at one significant match to an existing gene model in Blast-X search (Fig. 1c, Table 1 and Additional file 2: Table S2). Statistics results of eggNOG and GO classification of all annotated unigenes were shown in Additional file 3: Figure S1 and S2. 49.72% (*n* = 15,250) of homologs aligned to known proteins with sequence identify between 80 and 100%. Because the Tibetan naked carp was phylogenetically closer to zebrafish than some other fish species with complete genomic resources, we found 75.23% of the best hits (*n* = 23,074) were similar with model organism zebrafish (Additional file 4: Table S3). In addition, we extracted and aligned the putative CDSs in Tibetan naked carp transcriptome unigenes using TransDecoder, MAKER and ESTScan programs. Totally 93.95% (*n* = 28,817) of *G. przewalskii* unigenes with full length and partial CDSs were annotated (Table 1).

**Table 1:** Annotation results of *G. przewalskii* transcriptome unigenes

		Number	Percentage
Functional annotations	Total	28,519	92.98%
	Swiss-Prot	26,595	86.71%
	KEGG	21,203	69.13%
	NR	28,490	92.89%
	GO	21,657	70.61%
CDS annotations	Total	28817	93.95%
	TransDecoder	25736	83.91%
	MAKER	2647	8.63%
	ESTScan	434	1.42%

### Genomic evolution

**Fig. 3:**
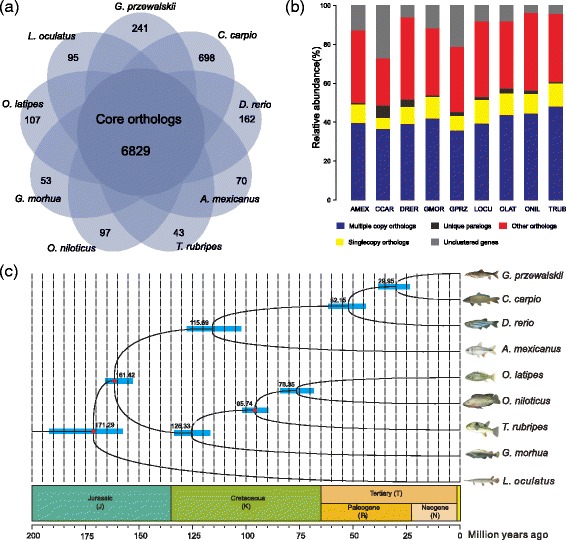
Comparison of genomic feature of *G. przewalskii* and other fish species. **a** Venn diagram showed shared and distinct gene families belonged to 9 fish species. The number of core orthologous genes within all species was 6,829. **b** Spinogram depicting the composition of different categories of gene families labeled by colors. AMEX, *Astyanax mexicanus*, CCAR, *Cyprinus carpio*, DRER, *Danio rerio*, GMOR, *Gadus morhua*, GPRZ, *Gymnocypris przewalskii*, LOCU, *Lepisosteus oculatus*, OLAT, *Oryzias latipes*, ONIL, *Oreochromis niloticus*, TRUB, *Takifugu rubripes*. **c** Divergence time estimation. The node bars indicate 95% posterior probability intervals. The red dots correspond to calibration points, and the specific calibration time was indicated in Methods section

A total of 213,853 proteins from *G. przewalskii* (*n* = 28,817) and eight other fish species, including *A. mexicanus*, *C. carpio*, *D. rerio*, *G. morhua*, *L. oculatus*, *O. latipes*, *O. niloticus*, *T. rubripes*, were binned into 30,211 orthologous groups (gene family) using OrthoMCL software following self-self-comparison with BLAST-P program. A total of 6,829 gene families were conserved among these nine fishes (Fig. 2a). Gene family expansion analysis showed that 214 gene families were expanded in *G. przewalskii* (Fig. 3a). Functional enrichment analysis suggested that significantly expanded gene families (*P* < 0.05) were involved in 131 GO categories of three main groups (Additional file 5: Table S4). The first group was related to metabolic process, such as cGMP metabolic process (GO:0046068, *P* = 0.00725), malate metabolic process (GO:0006108, *P* = 0.0000088) and one-carbon metabolic process (GO:0006730, *P* = 0.000081). The second largest group was associated with transport function, including water transport (GO:0006833, *P* = 0.00456), response to pH (GO:0009268, *P* = 0.0072), monovalent inorganic cation transport (GO:0015672, *P* = 0.000031). These data indicated that gene expansion was associated with high salinity and alkalinity environment in Lake Qinghai. Developmental functional category was the third group, such as pharyngeal muscle development (GO:0043282, *P* = 0.00576), heart trabecula formation (GO:0060347, *P* = 0.0000092). By comparing the orthologous groups between nine fish species, we identified 28,817 *G. przewalskii* genes clustered into 15,574 gene families (Table 2). These results indicated that gene models of *G. przewalskii* were similar to those of other representative well-annotated vertebrates.

**Table 2:** Summary of orthologous groups among nine species

Species	Number of genes	Unclustered	Genes in families	Number of families	Average genes per family
*A. mexicanus*	23,008	2,948	20,060	14,401	1.393
*C. carpio*	39,140	10,562	28,578	13,749	2.079
*D. rerio*	25,355	1,525	23,830	15,189	1.569
*G. morhua*	19,821	2,296	17,525	12,929	1.355
*G. przewalskii*	28,817	6,092	22,725	15,574	1.459
*L. oculatus*	18,304	1,481	16,823	13,260	1.269
*O. latipes*	19,531	1,559	17,972	12,901	1.393
*O. niloticus*	21,422	785	20,637	13,760	1.500
*T. rubripes*	18,455	768	17,687	12,615	1.402

### Phylogeny inference and divergence time estimation

Among 6,829 shared core orthologs, we identified 2,178 putative single-copy genes (only one orthologs in each gene family) in each fish species (Fig. 3b), making them suitable for phylogenetic inference and divergence time estimation. In order to maximize the information content of sequences and minimize the impact of missing data, stringent criterion was used to filter 2,178 single-copy orthologous groups with stricter constraints, including length (minimum 200 aa), sequence alignment (maximum missing data 50% in CDS alignments). We eventually obtained 1,159 groups and concatenated them into a single supergene for each fish species using a custom Perl script. Each of which was then subjected to phylogeny analyses with in PhyML 3.1 software [36]. Phylogenetic tree based on 1,159 individual nuclear genes was supported with 100% bootstrap values, consistent with the tree on mitochondrial genes or nuclear DNA markers (Additional file 3: Figure S3).

All of the estimated divergent times were labeled on nodes of this phylogenetic tree (Fig. 3c), and were well-matched to data deposited in TIMETREE [38]. The molecular-clock approached predicted divergence between *G. przewalskii* and *C. carpio* lineages was 29.95 million years ago (Mya) with confidence interval 27.25 to 45.65 Mya (Fig. 3c).

### Accelerated evolution on Tibetan naked carp lineage

**Fig. 4:**
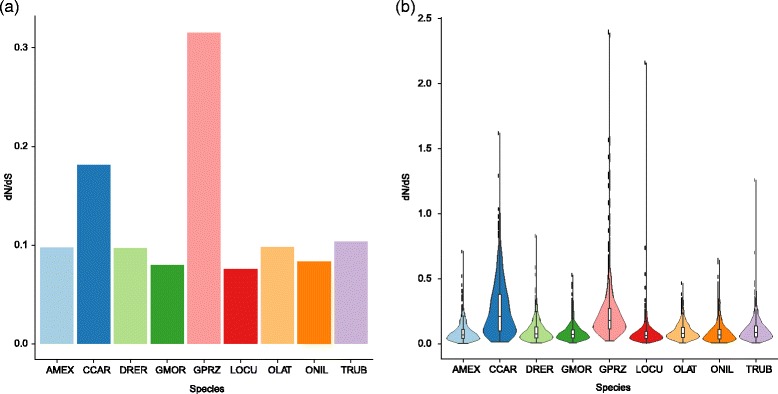
Comparison of selection feature of *G. przewalskii* and other fish species. **a** Average dN/dS ratios of concatenated all orthologs in *G. przewalskii* and other 8 fish species estimated by branch model in *codeml* program in PAML. **b** Violin plot showed the dN/dS ratios of each orthologous genes in 9 fish species estimated by branch-site mode

Adaptive divergence at molecular level may be reflected by an increased rate of non-synonymous changes within genes involved in adaptation [2]. We used a branch model constructed in PAML software to determine dN, dS, and dN/dS ratio values across all shared 6,829 orthologs in nine fish lineages. The higher dN/dS ratio in *G. przewalskii* lineages (with *P* < 2.2 × 10^−16^ in Wilcoxon rank sum test) implied that accelerated function evolution in *G. przewalskii* (Fig. 4a). Additionally, we analyzed the dN/dS ratio for each branch for a concatenated alignment of all 6,829 orthologs and 1,000 concatenated alignments constructed from ten randomly chosen orthologs. Intriguingly, using both comparison strategies, we found that *G. przewalskii* exhibited a significantly higher dN/dS ratio than eight other fish branches in present study (*P* < 2.2 × 10^−16^). These findings implicated that *G. przewalskii* was experienced the ongoing accelerated evolution under extreme environment (Fig. 4b).

### Fast evolving (FEG) and positively selected genes (PSG)

Using a set of 2,183 single-copy gene families retrieved from OrthoMCL, we conducted fast evolving and positive selection analyses to discover genes under the selection. In total, 513 FEGs were identified in *G. przewalskii* (Additional file 6: Table S5). Functional enrichment (GO) analysis suggested that these FEGs were enriched into energy metabolism, immune response, and transport functions. In the first group, many FEGs were related to mitochondrion, ATP binding and oxidative phosphorylation, such as NDUB9, COX11, MDH, ATP5c1 and ATP5b. In addition, we also identified a large number of immune genes, such as IRF8, C1q, C2, TNF10 which was involved in the stress responses as well. The last but most important finding was that FEGs included genes functioning in transports and ion channel (Additional file 6: Table S5), such as solute carrier (SLC) family (SLC9A3 and SLC19A3) and transmembrane protein (TM) family (TM9, TM33, TM97, TM120, TM175). Positive selection of these genes may provide the genetic basis for rapid adaptation to high saline and alkaline adaptation and tolerance.

Positive selection analysis pinpointed genes that were associated with a functional and environmental shift [41]. The branch-site model in PAML software identified 73 positively selected genes in *G. przewalskii* (Additional file 7: Table S6) that were possibly influenced the adaptation to high altitude aquatic life. GO analysis results indicated that PSGs had similar GO categories as FEG. For instance, 3 PSGs, PRKACA, ITPKA and PIGH were significantly enriched in energy metabolism. NKAP encodes NF-kappa-B-activating protein and TNFR1 encodes tumor necrosis factor receptor 1 were both related to immune response function. In addition, one PSG, SLC4A1 in SLC super family was also identified to be under positive selection. While comparing with published candidate PSGs identified from Tibetan wildlife [2–4, 42], we failed to detect any PSG potentially involved in hypoxia response function in *G. przewalskii*.

## Discussion

Comparative genomics analyses have been widely used to unveil the genetic basis of speciation [43–45] and adaptation of wild organisms [2, 3, 9, 10, 42]. Given the high expense of genome sequencing and complicated algorithm for genome assembly, particularly in polyploidy creatures, such as Schizothoracinae fish. Transcriptome sequencing is an effective and affordable approach to initiate comparative genomic analyses in non-model organisms. It mainly focuses on a large number of protein coding genes under natural selection. Here we sequenced and assembled multiple tissue transcriptome from *G. przewalskii* We comprehensively annotated this large-scale transcriptomic resources and identified up to 7,000 pairwise orthologs among nine fish genomes for the basis of comparative genomics analysis and functional verification. By conducting the comparative transcriptomic analysis, we treated *G. przewalskii* as a genomic resource to improve our understanding of the genetic makeup of fish species in the TP and to identify candidate genes underlying adaptation to the Tibetan Plateau of Schizothoracinae fishes.

### Sequencing information comparison

Although our present study based on five merged tissues libraries data seemed to provide less unigenes than previous report of gill and kidney transcriptome data, two another important parameters (N50 and average length of transcript) were even larger than a previous study (3,076 vs. 1,836; 1,988 vs. 952) [10]. We obtained a set of higher quality data and it was more appropriate for further comparative genomic analyses. For the first time, we comprehensively compared this reference transcriptome of *G. przewalskii* and eight other fish genome data from Ensemble database to identify expanded gene family, fast evolving and positively selected genes (PSG). Only PSG were identified in both subspecies of *G. przewalskii* [10]. The present study largely enlarged our understanding of adaptive strategies of *G. przewalskii* under extreme environment in the TP.

### Evolutionary history and speciation

*G. przewalskii* is the newly formed fish species in family Schizothoracinae during the separation of Lake Qinghai from the Yellow River [10, 12, 16, 18]. Based on mitochondrial genomes, evidence suggested that the split of two cyprinid fish *G. przewalskii* and *C. carpio* occurred approximately 68 mya in accordance with the early uplift of the TP around 50 mya [5]. Our genomic study support the notion that both cyprinid fish split at around 29.95 mya based on a larger number of single-copy orthologs. In addition, we reconstructed the phylogenetic tree of *G. przewalskii* with 8 other fish species with 100% bootstrap values, much more precise than previous studies using several mitochondrial or nuclear genes [46, 47]. Our results also implicated that high efficiency of transcriptomic data for phylogeny construction and accuracy of the divergence time estimation. Meanwhile, current study demonstrated that evolution of *G. przewalskii* may be driven by formation of extremely environmental conditions accompanied by the uplift of the TP.

### Elevated energy metabolism

Genome-wide studies on Tibetan terrestrial animals suggested that an increased evolutionary rate and positive selection on genes involved in energy metabolism, which contributed to highland adaptation [2–4, 9]. Our present work disclosed the adaptive strategy of Tibetan aquatic animals. Similar to Tibetan wild yak [2] and ground tit [3], gene families involved in metabolic processes were remarkably expanded in *G. przewalskii*, indicating the development of strong capacity to meet high energy demands in long-term low temperature aquatic environment. Environmental challenge tended to trigger gene duplication and neofunctionalization, new members in gene families possibly enhanced energy production efficiency in *G. przewalskii* by acquiring novel functions, which revealed by many cases [48–51]. In addition, genes showing signature of adaptive evolution in *G. przewalskii* also were involved into energy metabolism. Consistent with previous finding in Tibetan animals [2, 3], genes functioning in energy supply and ATP synthesis, such as NDUB9, encoding NADH ubiquinone oxidoreductase subunit B9 [52] and ATP5b, ATP synthase subunit beta [52, 53] were under strong positive selection in *G. przewalskii*.

### The adaptive evolution of immune genes in Tibetan naked carp lineage

Another adaptation of *G. przewalskii* to high altitude aquatic life in the TP may be the rapid evolution of immune genes, many of which were associated with innate immune system. Four FEGs (IRF8, TNF10, C1q and C2) and two PSGs (NKAP and TNFR1) were all involved into toll like receptor signaling pathway in innate immunity, which was identical to our previous findings [11, 15]. In addition, a recent study suggested that *G. przewalskii* was susceptible to infectious disease with high mortality in farming industry [54]. Another evidence showed that low diversity of pathogens occurred in Lake Qinghai of hypersaline and alkaline environment by previous survey [16, 17], which indicated that *G. przewalskii* survived in a lighter pathogen load environment. Therefore, we speculated that immune genes of *G. przewalskii* have experienced adaptive evolution and functional shifts to well adapted to this specific aquatic environment. Innate immune played an important role in fish to rapidly eliminate pathogen as the first line of defense against pathogen invasion, including bacteria and parasite [55, 56]. Recently, a large number of immune genes were identified in miiuy croaker and large yellow croaker to undergo adaptive evolution, which contributed to the fish well-developed immune defense pathogens and adaptation to dynamic aquatic environments [57, 58]. Compared to these studies, we also identified a number of immune genes showed signals of positive selection. Therefore, it was possible that adaptive evolution acting on innate immune genes in *G. przewalskii* to response to a lighter pathogen load in high salinity and alkalinity environment in Lake Qinghai.

### Expansion and adaptive evolution in transport function genes

We identified expanded gene families functioned in water transport, response to pH and monovalent inorganic cation transport in *G. przewalskii*. This result was consistent with findings in Amur ide (*Leuciscus waleckii*) that also survived in an extremely alkaline environment in Lake Dali Nur [59]. The alkaline environment of both Lake Qinghai and Lake Dali Nur spurred evolution and expansion of genes in transport function. SLC family was the largest common group identified by both groups, it codes transmembrane transporters for inorganic ions, amino acids, neurotransmitters, sugars, purines and fatty acids, and other solute substrates [60]. Recent evidences indicated that adaptive evolution of SLC family genes contributed to the response to salinity and alkalinity stress to fishes [59, 61]. SLC4 subfamily encodes bicarbonate-transporter and regulated of Cl^−^-HCO_3_^−^ exchange, playing critical roles in maintenance of intracellular pH equilibrium [62, 63]. SLC9 subfamily was essential for the regulation of Na^+^/H^+^ exchange [64]. Our study identified PSGs in SLC family, including SLC4A1, SLC9A3 and SLC19A3, which may acquire functional shift of transport to cope with the severe saline and alkaline stress in the Lake Qinghai.

### Hypoxia response and controversial issue

Low oxygen is a typical limiting factor for all the Tibetan terrestrial wildlife [5, 65]. A couple of candidate genes were identified to participate into the hypoxia response in Tibetan terrestrial animals, providing the genetic foundation for the adaptation to low oxygen levels [2–4, 7, 8]. However, the hypoxic environment and hypoxia response were the debatable topic for Tibetan aquatic animal [16–18]. Although previous studies demonstrated strong positive selection on genes related to hypoxia response in highland fishes [42, 66], we were unable to identify any FEGs and PSGs involved into hypoxia response in the present study. This difference could be explained by relatively high dissolved oxygen levels in Lake Qinghai compared to other highland lakes, resulting from abundant and diverse of hydrophyte species [16, 17]. The comprehensive ecological and genomic analyses were both required to confirm the hypoxia environments and the potential hypoxia response in *G. przewalskii*.

## Conclusions

Tibetan naked carp *G. przewalskii* exhibits a spectacular adaptation to extreme cold, high saline and alkaline aquatic environment in Lake Qinghai. It serves as a remarkable model to understand evolutionary scenarios occurring under environmental changes during the uplift of the TP. In current study, we generate a reference transcriptome of *G. przewalskii* and provide an important genetic resource for comprehensive comparative genomic analyses across teleost fish. Our results suggest that gene families predominantly expanded in energy metabolism and transport function in *G. przewalskii*. The potential neofunctionalization of novel genes may contribute to the adaptation to the extreme environment in Lake Qinghai. Adaptive evolution occured in genes involved into metabolism, immune system and transport functions, and reinforcements the functional adaptation to the chronic cold, extreme alkaline and saline, lighter load of pathogens environment in Lake Qinghai. Additionally, the current study also shed lights on the functional validation of candidate genes contributed to extreme environment adaptation.

## Additional files

Additional file 1: Table S1. Summary of sequencing, assembly and analysis of *G. przewalskii* transcriptome. (DOCX 15 kb)
Additional file 2: Table S2. Annotation of *G. przewalskii* unigenes. (XLSX 4492 kb)
Additional file 3: Figure S1. eggNOG classification of *G. przewalskii* transcriptome. **Figure S2**. GO classification of *G. przewalskii* transcriptome. **Figure S3**. Phylogenetic tree of *G. przewalskii* and 8 fish species. (DOCX 3527 kb)
Additional file 4: Table S3. Species distribution information. (DOCX 13 kb)
Additional file 5: Table S4. Expansion gene family and annotation. (XLSX 15 kb)
Additional file 6: Table S5. Fast evolved gene (FEGs) list. (XLSX 40 kb)
Additional file 7: Table S6. Positively selected genes (PSGs) list. (XLSX 13 kb)
