# Supplementary material for: Comprehensive transcriptomic analysis of Tibetan Schizothoracinae fish Gymnocypris przewalskii reveals how it adapts to a high altitude aquatic life
Source: BMC Evol Biol. 2017 Mar 9;17:74. doi: 10.1186/s12862-017-0925-z (PMC5343388; doi:10.1186/s12862-017-0925-z)
Supplement: Additional file 1: Table S1. — Summary of sequencing, assembly and analysis of G. przewalskii transcriptome. (DOCX 15 kb) [file 12862_2017_925_MOESM1_ESM.docx]

**Table S1. Summary of sequencing, assembly and analysis of *G. przewalskii* transcriptome.**

| Dataset name | All | Gill | Kidney | Heart | Liver | Brain |
| --- | --- | --- | --- | --- | --- | --- |
| Total bases (bp) | 36,321,889,441 | 7,074,500,220 | 7,234,421,400 | 7,317,914,220 | 7,379,639,030 | 7,315,414,571 |
| **No. of reads** |  |  |  |  |  |  |
| Raw reads | 445,582,631 | 85,371,306 | 88,787,918 | 91,512,202 | 89,460,104 | 90,451,101 |
| Clean reads | 404,479,795 | 80,382,460 | 78,605,558 | 82,521,158 | 81,984,878 | 80,985,741 |
| Q20^a^ of clean reads | 97.36% | 97.22% | 97.64% | 97.31% | 97.23% | 97.39% |
| **No. of contigs** |  |  |  |  |  |  |
| Total contigs | 1,236,939 | 239,166 | 240,762 | 243,457 | 256,375 | 257,179 |
| Average contig read length (bp) | 310 | 316 | 307 | 312 | 302 | 311 |
| **No. of unigenes** |  |  |  |  |  |  |
| Total unigenes | 30,672 | 132,554 | 130,604 | 135,634 | 134,156 | 133,471 |
| N50^b^ of unigenes (bp) | 3,076 | 1,490 | 1,399 | 1,557 | 1,532 | 1,536 |
| Average unigene read length (bp) | 1,988 | 736 | 704 | 748 | 745 | 747 |

a Q20: percentage is the proportion of nucleotides with a quality value > 20 in reads.

b N50: unigene length-weighted median.
