# Supplementary material for: Comprehensive transcriptomic analysis of Tibetan Schizothoracinae fish Gymnocypris przewalskii reveals how it adapts to a high altitude aquatic life
Source: BMC Evol Biol. 2017 Mar 9;17:74. doi: 10.1186/s12862-017-0925-z (PMC5343388; doi:10.1186/s12862-017-0925-z)

**Supplementary figure**

**FIG. S1.** eggNOG classification of *G. przewalskii* transcriptome.


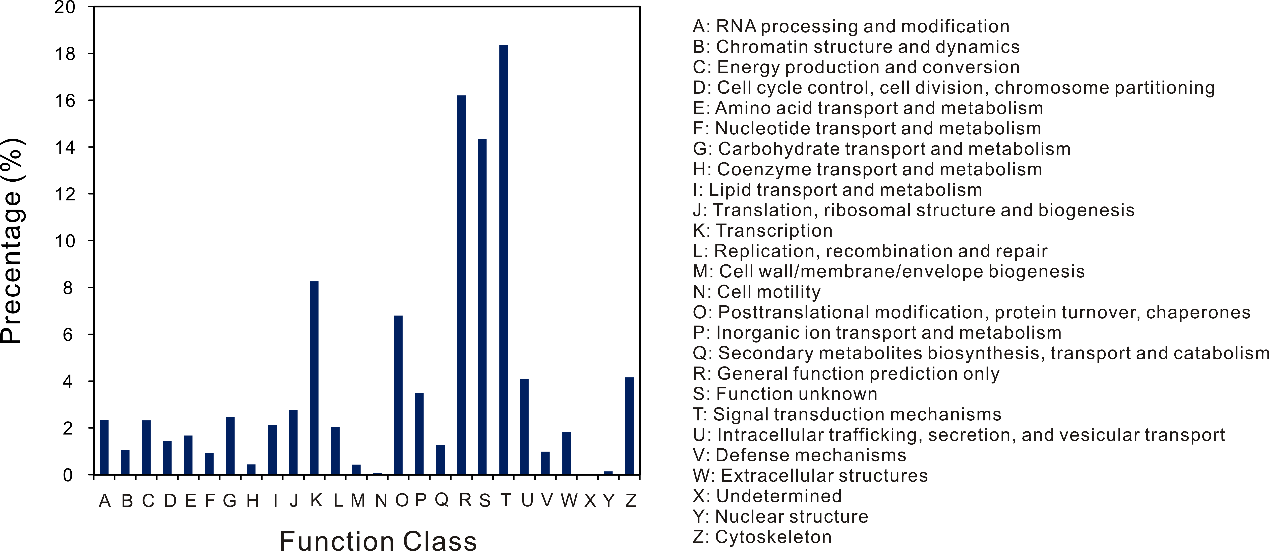


**FIG. S2.** GO classification of *G. przewalskii* transcriptome.


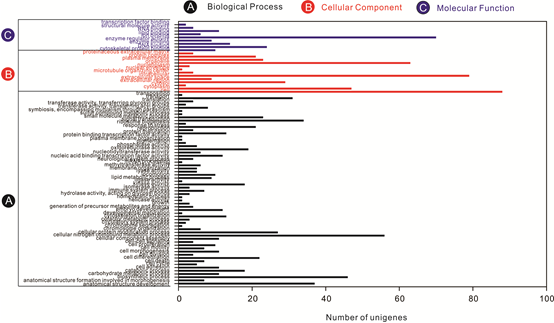


**FIG. S3.** Phylogenetic tree of *G. przewalskii* and eight fish species.


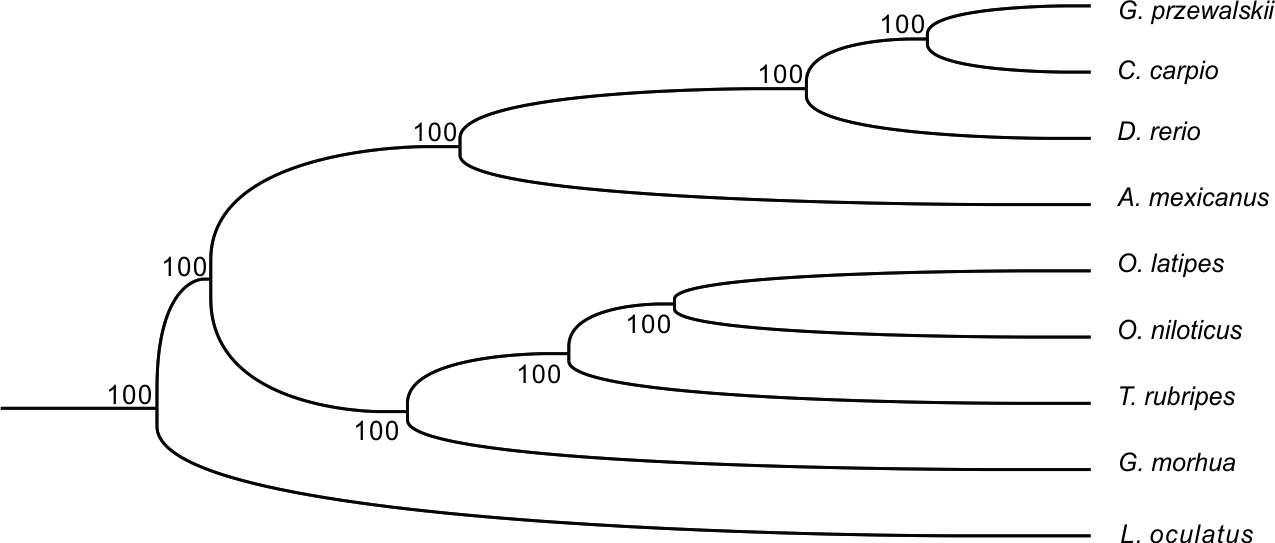

Supplement: Additional file 3: Figure S1. — eggNOG classification of G. przewalskii transcriptome. Figure S2. GO classification of G. przewalskii transcriptome. Figure S3. Phylogenetic tree of G. przewalskii and 8 fish species. (DOCX 3527 kb) [file 12862_2017_925_MOESM3_ESM.docx]
