# Supplementary material for: Comprehensive transcriptomic analysis of Tibetan Schizothoracinae fish Gymnocypris przewalskii reveals how it adapts to a high altitude aquatic life
Source: BMC Evol Biol. 2017 Mar 9;17:74. doi: 10.1186/s12862-017-0925-z (PMC5343388; doi:10.1186/s12862-017-0925-z)
Supplement: Additional file 4: Table S3. — Species distribution information. (DOCX 13 kb) [file 12862_2017_925_MOESM4_ESM.docx]

**Table S3.** Species distribution

| Species name | Number of unigenes | Percentage |
| --- | --- | --- |
| *Danio rerio* | 23,074 | 75.23% |
| *Maylandia zebra* | 1,662 | 5.42% |
| *Oreochromis niloticus* | 1,091 | 3.56% |
| *Oryzias latipes* | 812 | 2.65% |
| *Takifugu rubripes* | 555 | 1.81% |
| *Salmo salar* | 429 | 1.40% |
| *Tetraodon nigroviridis* | 352 | 1.15% |
| other | 2,693 | 8.78% |
